# Supplementary material for: CATransU-Net: Cross-attention TransU-Net for field rice pest detection
Source: PLoS One. 2025 Jun 25;20(6):e0326893. doi: 10.1371/journal.pone.0326893 (PMC12194106; doi:10.1371/journal.pone.0326893)
Supplement: Attachment — Submitted filename: renamed_7e15a.docx [file pone.0326893.s001.docx]

**Research evaluation**

**CATransU-Net: Cross-Attention TransU-Net for Field Rice Pest Detection**

**Research Summary**

The paper proposes **CATransU-Net**, a hybrid deep learning model combining U-Net and Transformer architectures for rice pest detection in field environments. Key innovations include:

- **Dilated Residual Inception (DRI)** for multiscale feature extraction.
- **Dual Transformer-Attention (DTA)** to integrate local and global contextual information.
- **Cross-Attention Skip-Connection (CASC)** for enhanced encoder-decoder feature fusion.

The model is validated on the IP102 dataset, achieving **93.51% precision**, outperforming U-Net (84.15%) and other state-of-the-art methods. The work addresses challenges like pest size variability, background complexity, and intra-class similarity in field conditions.

### Key Contributions

1. **Hybrid Local-Global Feature Learning**:
   - DRI captures multiscale pest features, while DTA integrates global context, addressing intra-class variations.
2. **Contextual Feature Fusion**:
   - CASC replaces traditional skip-connections, preserving spatial and semantic details during upsampling.
3. **Benchmark Performance**:
   - Achieves **9.36% higher precision than U-Net**, validating the efficacy of Transformer-CNN integration.

### Strengths

1. **Innovative Architecture**:
   - Combines U-Net’s local feature extraction with Transformer’s global context modeling, addressing both small-scale and irregular pests.
   - CASC effectively bridges encoder-decoder features, reducing information loss during upsampling.
2. **Robust Evaluation**:
   - Comprehensive ablation studies validate the contributions of DRI, DTA, and CASC modules.
   - Benchmarked against six models (e.g., TransUnet, DMSAU-Net), demonstrating superior performance.
3. **Practical Relevance**:
   - Targets real-world challenges (e.g., complex backgrounds, occlusions) with a focus on field applicability.
   - Open-source code and dataset accessibility enhance reproducibility.
4. **Theoretical Soundness**:
   - Detailed explanations of modules (e.g., TSA, GSA) and loss functions provide clarity on model mechanics.

### Weaknesses

1. **Computational Overhead**:
   - Transformer components increase training time (1.64 hours vs. U-Net’s 1.01 hours), limiting real-time deployment potential.
2. **Dataset Limitations**:
   - Class imbalance in IP102 (e.g., some pests have only 173 images) may bias results.
   - Limited testing on other datasets reduces generalizability claims.
3. **Implementation Gaps**:
   - No discussion of inference speed or hardware requirements for field deployment.
   - Lightweight design (mentioned in future work) is not explored, despite its importance for edge devices.
4. **Comparison Breadth**:
   - Omit recent models (e.g., Mask2Former, ConvNeXt) that could provide stronger baselines.

### Suggestions for Improvement

1. **Address Class Imbalance**:
   - Use data augmentation (e.g., SMOTE, GANs) or re-sampling techniques to mitigate bias in IP102.
2. **Optimize for Real-Time Use**:
   - Explore model compression (e.g., pruning, quantization) or lightweight Transformers (e.g., MobileViT).
3. **Expand Comparisons**:
   - Include recent architectures like Mask R-CNN, Swin Transformer, or Vision Transformers (ViTs).
4. **Field Deployment Analysis**:
   - Evaluate inference speed on edge devices (e.g., drones, IoT sensors) and discuss computational trade-offs.
5. **Generalizability Testing**:
   - Validate the model on diverse datasets (e.g., Pest24, AgriPest) to assess cross-domain performance.
6. **Ethical Considerations**:
   - Discuss potential biases (e.g., rare pest detection) and environmental impacts of automated pest control.

### Evaluation Notes

- **Visualization Quality**: Figures 6–9 provide clear qualitative comparisons but lack quantitative metrics (e.g., IoU scores).
- **Reproducibility**: Code availability is a strength, but dependencies (e.g., PyTorch version) should be explicitly documented.
- **Impact on Agriculture**: Emphasize how CATransU-Net could reduce pesticide overuse through precise pest detection.

### Conclusion

The paper presents a well-designed solution to rice pest detection with strong theoretical and empirical contributions. While computational efficiency and dataset limitations need addressing, the integration of CNNs and Transformers sets a promising direction for agricultural AI. Future work should prioritize lightweight deployment and broader ecological validation.

**Latakia, 12/02/2025**

**Reviewer**

**Prof. Dr. Nabil ABO KAF**
